# First District Dental Society of New York

**Published:** 1887-02

**Authors:** 


### FIRST DISTRICT DENTAL SOCIETY OF NEW YORK.

REPORTED EXPRESSLY FOR THE INDEPENDENT PRACTITIONER.

The Society held a regular monthly meeting on the evening of January 3, 1887, at the rooms of the S. S. White Dental Manufacturing Company, corner of 32d street and Broadway, the President, Dr. Wm. Carr, in the chair. After some routine business and the election of three new members, the report of the special meeting of this society, held November 19, 1886, was taken from the table and read by the secretary.

Dr. Abbott inquired why the Kingsley resolutions had been left out, when the President declared them out of order.

Dr. Abbott then moved that the secretary of the society be instructed to officially correct a statement concerning them, which appeared in the December number of the INDEPENDENT PRACTITIONER, page 712. The motion was carried.

The President then called for the report of the clinic committee, which was presented by the chairman, Dr. C. F. W. Bödecker, as follows:

The attendance was between sixty and seventy. Dr. Evans exhibited and explained some gold crowns, the most natural in appearance that have ever been presented at the clinic. The grinding surfaces were made by the method of G. W. Melott, using moldine and the Melott fusible metal for making the die.

Dr. Evans also exhibited some crowns with a porcelain face, which can be used over living pulps.

Dr. W. J. Thayer, of Brooklyn, filled a right upper second bicuspid on the mesial and grinding surfaces. Kearsing's No. 4 soft foil, rolled into a rope and cut as required, was employed, and was introduced by means of his new mechanical trip mallet, which, however, was broken during the operation, the filling being finished with a lead mallet.

Dr. Lawrence Vanderpant, of Orange, N. J., exhibited a geoplastic lower set of artificial teeth with a pink rubber front, made by his process. The teeth used were Ash's diatoric.

Dr. G. F. Reese, of Brooklyn, exhibited an upper set of teeth, with gum blocks made of Reese's metal.

Dr. Crowell exhibited a partial upper continuous gum set.

The President then called upon Dr. Frank Abbott, the regular assayist of the evening, who read a paper upon the structure of the enamel. It was principally a corroboration of what had been previously published by Drs. Heitzman and Bödecker, but the microscopical specimens (of which drawings were shown) had been prepared in the manner advised by Dr. G. W. Weld, to whom Dr. Abbott gave credit for the discovery of the new method, which was by the decalcification of ground sections of enamel in a six per cent. solution of acetic acid. On these specimens the enamel fibers of living matter were claimed to be so plainly visible that no honest observer could doubt their presence, and not only was this the case with the enamel fibers and their coarse offshoots, but it was also true of the minute network present in the enamel prisms.

The President, announcing the subject open for discussion, called upon Dr. Carl Heitzman, who drew attention to the remarkable fact that in spite of Dr. Bödecker's discovery some eight years ago, that enamel is a living tissue, there are still those who assert that it is nothing but a "coat of mail." This view is held by Dr. Sudduth in the "System of American Dentistry." The method of Dr. Weld, it is to be hoped, will at last remove all doubts as to the nature of the enamel, since it enables us to decalcify and prepare it for microscopical examination in a comparatively short time, and without any difficulties, as regards technicalities.

Dr. Heitzman desired to lay stress upon the assertion of Dr. Abbott, that there is no interlacing of bundles of enamel prisms, and upon the fact that some bundles run a more wavy course than others, which explain the occurrence of transverse sections of enamel prisms between longitudinal sections. He also admits the correctness of the view of Dr. Abbott, that the enamel prisms are quite frequently supplied at their borders with a ledge, resisting the action of acetic acid, and being, probably, elastic substance, similar to that surrounding the dentinal canaliculi. Both the clinical and microscopical features are such that we could expect the acceptance of the view by all scientific dentists, that enamel is a tissue, endowed with properties of life.

Prof. Andrews, of Boston, was not ready to discuss the paper, although he had seen the specimens. He was sorry to hear Dr. Heitzman denounce the work of Drs. Williams and Sudduth.

Dr. Allen was not convinced of the presence of enamel fibers in the enamel, but admitted that it contained some organic matter.

Dr. F. Y. Clark fully agreed with the views advanced in the paper, and was surprised that any close observer could doubt that enamel was alive. Any one can demonstrate this for himself by eating sour fruits, such as lemons or grapes, the juices of which dissolve a portion of the lime-salts of the enamel, exposing the ends of the living matter.

Dr. C. F. W. Bödecker thanked Dr. Abbott for his valuable paper, as well as Dr. Heitzman for his assistance while searching for enamel fibers about eight years ago ; he had made use of almost all acids, except acetic, with the view of softening the enamel sufficiently to cut with a razor, but was not successful, He then resorted to the process of grinding freshly extracted teeth under water, a process in which Dr. Heitzman had no confidence whatever, his prejudices being so great that he refused to look at specimens prepared in this manner, until on the 6th of July, 1878, Dr. Bödecker succeeded in staining the enamel fibers with chloride of gold. He had shown such specimens of enamel to many of his medical friends who were histologists, but every one expressed the opinion that the fibers in the enamel were protoplasm. Dr. Bödecker thought that the opposition to the acceptance of his views was mainly due to prejudice. He had showed to Dr. Waldstein, a former assistant of Prof. Arnold, of Heidelberg, specimens of the development of enamel, and he acknowledged that the stellate reticulum seen was a myxomatous connective tissue, but would not call it by that name, as it was not in accordance with the present views of physiology, although he could not explain why it should not be called connective tissue.

Dr. Wm. H. Atkinson was one of the first dentists who attended at Dr. Heitzman's laboratory. He was very glad that this opportunity had been offered him, but was sorry that he had not been so situated that he could continue the studies. He had sometimes differed with Heitzman, but this did not detract anything from his admiration for his work.

Dr. Atkinson believed that if the System of Dentistry, published by Lea Brothers & Co., were condensed to one-eighth its present volume, it would do some good to the profession. The work was well printed and bound, and that was the best part of it. He believed it was got up more for selling than for anything else.

